# Performance of Simplexa Dengue Molecular Assay Compared to Conventional and SYBR Green RT-PCR for Detection of Dengue Infection in Indonesia

**DOI:** 10.1371/journal.pone.0103815

**Published:** 2014-08-07

**Authors:** R. Tedjo Sasmono, Aryati Aryati, Puspa Wardhani, Benediktus Yohan, Hidayat Trimarsanto, Sukmal Fahri, Tri Y. Setianingsih, Febrina Meutiawati

**Affiliations:** 1 Eijkman Institute for Molecular Biology, Jakarta, Indonesia; 2 Clinical Pathology Department, School of Medicine and Institute of Tropical Disease, Airlangga University, Surabaya, Indonesia; 3 Agency for the Assessment and Application of Technology, Jakarta, Indonesia; 4 Health Polytechnic, Jambi Provincial Health Office, Kotabaru, Jambi, Indonesia and Graduate School in Medicine, Diponegoro University, Semarang, Indonesia; University of Hong Kong, Hong Kong

## Abstract

Diagnostic tests based on detection of dengue virus (DENV) genome are available with varying sensitivities and specificities. The Simplexa Dengue assay (Focus Diagnostics) is a newly developed real-time RT-PCR method designed to detect and serotype DENV simultaneously. To assess the performance of the Simplexa Dengue assay, we performed comparison with conventional RT-PCR and SYBR Green real-time RT-PCR on patients sera isolated from eight cities across Indonesia, a dengue endemic country. A total of 184 sera that were confirmed using NS1 and/or IgM and IgG ELISA were examined. Using conventional and SYBR Green real-time RT-PCR, we detected DENV in 53 (28.8%) and 81 (44.0%) out of 184 sera, respectively. When the Simplexa Dengue assay was employed, the detection rate was increased to 76.6% (141 out of 184 samples). When tested in 40 sera that were confirmed by virus isolation as the gold standard, the conventional RT-PCR yielded 95% sensitivity while the sensitivity of SYBR Green real-time RT-PCR and Simplexa Dengue assay reached 97.5% and 100%, respectively. The specificities of all methods were 100% when tested in 43 non-dengue illness and 20 healthy human samples. Altogether, our data showed the higher detection rate of Simplexa Dengue compared to conventional and SYBR Green real-time RT-PCR in field/surveillance setting. In conclusion, Simplexa Dengue offers rapid and accurate detection and typing of dengue infection and is suitable for both routine diagnostic and surveillance.

## Introduction

Dengue is the most important arthropod-borne viral infection of humans with a large global burden. There are an estimated 50 million infections per year occurring across approximately 100 countries in tropical and sub-tropical regions in the world with potential for wider distribution. The disease affects approximately 2.5 billion people living in Southeast Asia, the Pacific, and the Americas [1], [2]. Dengue disease causes varying clinical manifestations ranging from an undifferentiated fever (Dengue Fever, DF) to the more severe forms of the disease including Dengue Hemorrhagic Fever (DHF) and Dengue Shock Syndrome (DSS) [3].

Dengue disease is caused by dengue virus (DENV), a member of Flaviviridae family, with a substantial genetic diversity shown by the presence of four serotypes (DENV-1, -2, -3, and -4) and multiple genotypes (or subtypes) within each serotype [4], [5]. DENV is transmitted through a human-mosquito cycle with the aid of *Aedes aegypti* and *Ae. albopictus* mosquito vectors. The genome consists of single-stranded positive-sense RNA which encodes three structural (C, prM/M, E) and seven non-structural proteins (NS1, NS2A, NS2B, NS3, NS4A, NS4B, NS5) [1].

Laboratory confirmation of dengue is important since the broad spectrum of clinical presentations causes the difficulties in making an accurate diagnosis. The available dengue diagnostic tools employ the detection of dengue virus, viral antigen, dengue genome/RNA, and/or serology [6]. Serological detection is useful and is currently the most widely-used dengue diagnostic [6], [7] for dengue detection after 1 week of the fever [8], but it cannot be used earlier than 3–5 days after fever or for discriminating serotypes [9].

The molecular detection of DENV RNA genome is an alternative to the serological detection of the virus infection. This method offers a sensitive, rapid and simple mean for clinical diagnosis, and has been widely used in DENV research, including entomological surveillance and molecular epidemiological studies, dengue pathogenesis, antiviral drug and vaccine studies [7]. Because of their sensitivity, molecular techniques have gradually replaced traditional virus isolation method as the new standard for DENV detection in acute-phase serum samples [10]. Common methods in DENV nucleic acid detection include reverse-transcription and polymerase chain reaction (RT-PCR), one step or nested PCR, nucleic acid sequence-based amplification (NASBA), and real-time RT-PCR [10], [11].

Among the many available strategies for RT-PCR DENV detection, a two-step RT-PCR method developed by Lanciotti *et al.* [12] has been widely used. This method offers simple detection and typing of dengue viruses, especially in laboratory with limited resources. It utilizes consensus PCR primers that amplify the C and prM genes of dengue viruses. The D1 and D2 primers used in the first run of RT-PCR rapidly detect DENV genome, while the 4 serotype-specific primers TS1, TS2, TS3, and TS4 in the second run are used for serotyping by analyzing the unique sizes of the amplicons for each serotype [12]. This method is proven to be more sensitive than other available conventional RT-PCR methods [13].

A fluorescent-based real-time RT-PCR methods for dengue detection have been used increasingly and have better sensitivity and specificity compared to conventional RT-PCR [11]. One of the methods offers a cost-effective assay using the SYBR Green dye [14]. This method utilizes pan-dengue primers to detect all serotypes of DENV and was proven to be more sensitive than other methods [15].

Other methods of real-time based RT-PCR are also available and have been evaluated for their performance [16], [17].

A new generation of real-time RT-PCR for the detection and serotyping of dengue has been developed recently. The Simplexa Dengue (Focus Diagnostics) assay employs bi-functional fluorescent probe-primers and reverse primers to amplify NS5, NS3, NS5, and capsid genes of DENV. This new method has been successfully used for dengue detection both in human and mosquito vectors [18], [19]. Because of the limited information on performance of this method, we sought to determine the usefulness of this method in detecting dengue infection on patients recruited during our surveillance in eight cities in Indonesia. Comparison with the conventional RT-PCR and SYBR Green real-time RT-PCR was performed on antigen/serologically and virus isolation-confirmed dengue patients’ sera.

## Materials and Methods

### Ethics statement

Ethical clearances were obtained from Medical Research Ethics Committees of Airlangga University, Surabaya and Diponegoro University, Semarang, Indonesia. Samples from dengue patients were included in the study upon obtaining written informed consents from adult patients. For minors/children participants, written informed consents were sought from their parent/legal guardians.

### Sample collection and serological tests

A total of 184 dengue-positive clinical samples used in this study were collected from hospitals and health centers in eight provincial capital cities across Indonesian archipelago, namely Jakarta, Surabaya, Semarang, Medan, Denpasar, Kendari, Jayapura and Samarinda in 2010–2012. All samples were collected during acute phase, typically within the first five days of illness. Detection of DENV NS1 antigen was performed using Panbio Dengue Early ELISA (Alere, Brisbane, Australia), according to manufacturer’s instructions. To complement NS1 detection, the Panbio Dengue Duo IgM & IgG Capture ELISA (Alere) was also performed on all samples and used to determine the infection status (primary or secondary infection) according to manufacturer’s protocol. Briefly, the positive IgM result (>11 of Panbio Units) is indicative of active primary or secondary infection. IgG positive result (>22 Panbio Units) is indicative of active secondary infection. Primary infection was determined by positive IgM (>11 Panbio Units) and negative IgG (<22 Panbio Units) while secondary infection was determined by positive IgG (>22 Panbio Units) which may be accompanied by elevated IgM levels. For virus isolation, sera which were confirmed positive by NS1 ELISA were then cultured. Of those, 40 samples were successfully virus-isolated and E gene-sequenced. These samples were used as the gold standard for comparison of conventional RT-PCR, SYBR Green real-time RT-PCR, and Simplexa Dengue for method evaluation. A total of 43 sera from patients diagnosed as having non-dengue infection, i.e. typhoid (n = 24), leptospirosis (n = 1), measles (n = 1), pertussis (n = 1), malaria (n = 15) and bacterial septicemia (n = 1) confirmed by clinical and laboratory tests, were used as non-dengue cases control. Twenty healthy human samples were also included to test the specificity of the assays. The number of sample for dengue (n = 184) and non-dengue (n = 63) used in this study exceeded the required minimum sample size estimated by Conner’s formula for McNemar’s test in paired study design [20]. Assuming that the RT-PCR method has 75% sensitivity and 100% specificity versus the estimated 90% sensitivity and 85% specificity of Simplexa assay, with a 5% alpha and 20% beta errors, the minimum sample sizes for dengue and non-dengue are n = 102 and n = 49, respectively.

### RNA extraction

Strict controls on RNA extraction and PCR preparation/reaction procedures were employed to prevent cross-contamination between samples, in which activities were performed in separate areas/containments and using separate sets of equipment. For RT-PCR activity, reagent preparation and PCR amplification were performed in areas dedicated for each activity. All experiments were conducted in a GCLP-certified laboratory under the UK-Research Quality Association scheme [21] that ensures the compliance with Good Clinical Laboratory Practice.

Viral RNAs were extracted from serum samples using QIAamp Viral RNA Mini Kits (Qiagen, Hilden, Germany) or MagNA Pure LC Total Nucleic Acid Isolation Kit (Roche, Mannheim, Germany) performed in an automated MagNA Pure LC 2.0 Instrument (Roche) according to manufacturers’ instructions. Because of the limited volume of sera available, we were not able to extract all samples using both extraction methods. Extraction using the QIAamp Viral RNA Mini kit was performed for 134 samples, while extraction using MagNA Pure LC was performed for 50 samples. To ensure the uniformity of RNA samples between two methods of extraction employed, the same RNA sample from each dengue patient was assayed using conventional RT-PCR, SYBR Green real-time RT-PCR, and the Simplexa Dengue assay.

### Conventional RT-PCR

DENV nucleic acid detection and serotyping using conventional RT-PCR was done according to the two step protocol previously described by Lanciotti, et al [12], with modification according to Harris, et al. [22]. Dengue viral RNAs extracted as described above were reverse-transcribed into cDNA using Superscript III reverse transcriptase (RT) (Invitrogen-Life Technologies, Carlsbad, CA). Subsequently, cDNA was amplified using *Taq* DNA polymerase (Roche). The detection of DENV was facilitated by amplification of 511 bp PCR product which was then used as template for the DENV serotyping. The four dengue serotypes were distinguished by PCR product size upon electrophoresis of PCR products on 2% agarose gels.

### SYBR Green real-time RT-PCR using generic pan-dengue primers

Real-time RT-PCR assay was established according to the cost-effective real-time RT-PCR protocol to screen for dengue virus [14]. The assay uses generic pan-dengue primers targeting 3′-untranslated region conserved for all serotypes of DENV. The primer sequences were as follows: pan-dengue F (5′-TTGAGTAAACYRTGCTGCCTGTAGCTC-3′) and pan-dengue R (5′-GAGACAGCAGGATCTCTGGTCTYTC-3′). The one-step RT-PCR assay was performed using Superscript III Platinum SYBR Green One-Step qRT-PCR Kit (Invitrogen-Life Technologies), according to manufacturer’s recommendation. The reaction was prepared in a 96-well plate format, using 5 µl of RNA template and 200 nM of each primer in a final volume of 20 µl. The 5-carboxy-X-rhodamine (ROX) reference dye was used to normalize the fluorescent reporter signal according to manufacturer’s instruction. The reactions were allowed to run on an ABI 7500 real-time PCR machine (Applied Biosystems, Foster City, CA). Thermal cycle settings consist of a 10 minutes reverse transcription step at 50°C, followed by 5 minutes of *Taq* polymerase activation at 95°C, 40 cycles of 15 seconds denaturation step at 95°C and 45 seconds of annealing and extension steps at 60°C, continued with default melting curve analysis step. The fluorescence emitted was captured at the annealing and extension step of each cycle at 530 nm. Cycle threshold (Ct) value was determined as the cycle where the fluorescence of a sample increases to a level higher than the background fluorescence. No-template controls (NTCs) and dengue-positive RNAs extracted from DENV-1 WestPac, DENV-2 NGC, DENV-3 H87, and DENV-4 H241 were included in each assay run as controls. The melting temperature (Tm) of each PCR amplification product was checked to verify the correct products. Reactions with a high Ct value or ambiguous Tm value were analyzed by gel electrophoresis on a 2% agarose gel to confirm the presence of correct amplicon size. On this study, this method was used only for dengue detection and not for DENV serotyping.

### Simultaneous DENV detection and serotyping using Simplexa Dengue

**Figure 1:**
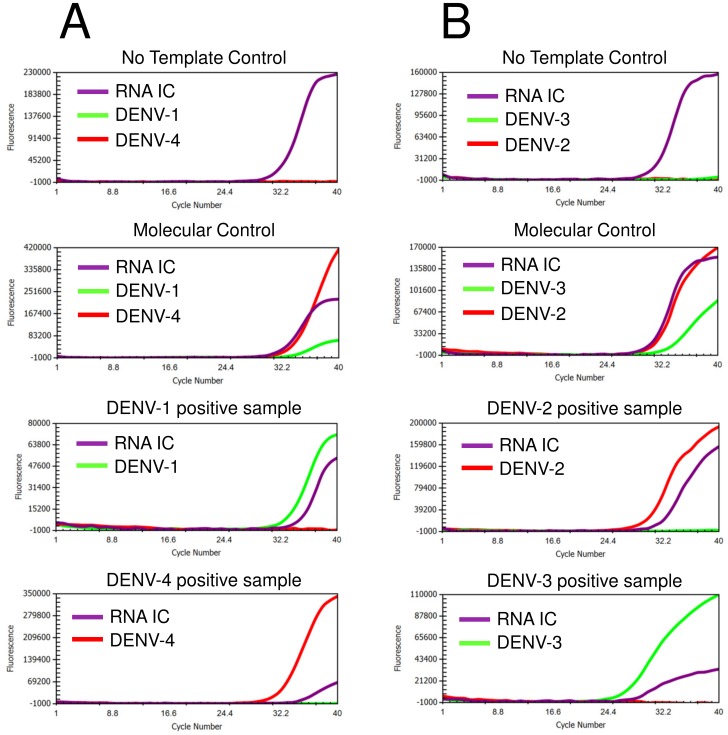
Representative of Simplexa Dengue real-time RT-PCR amplification curves of dengue detection. Detection reactions were separated in two tubes, i.e. DENV-1 and DENV-4 (A) and DENV-2 and DENV-3 (B). Assessment of results were based on the value of controls, which include the Molecular control (MC), RNA internal control (RNA IC) for extraction, and no-template control (NTC).

The same RNA samples were also subjected to dengue detection and serotyping using the Simplexa Dengue assay (Focus Diagnostics, Cypress, CA, USA). The assay is a real-time RT-PCR that discriminates DENV-1 and -4 in one reaction, and DENV-2 and -3 in another reaction. Bi-functional Scorpion-based fluorescent probe-primers together with reverse primers were used in this method to amplify NS5, NS3, NS5, and capsid genes of DENV-1, DENV-2, DENV-3, and DENV-4, respectively. An RNA internal control (RNA IC) is used to monitor the RNA extraction process and to detect RT-PCR inhibition. Briefly, two reaction mixes (1 & 4 and 2 & 3) were prepared according to manufacturer’s instructions. The mixes consist of serotype-specific primers mixes, *Taq* Polymerase, and RT enzyme. Five microliters of the reaction mixes were added into designated wells of Universal Disc (3M-Focus Diagnostics) followed by the addition of 5 µl RNA samples, Molecular Control (MC, consisted of inactivated dengue virus serotypes -1, -2, -3, and -4), and No Template Control (NTC). Each sample was tested using serotype 1 & 4 and 2 & 3 reaction mixes in different spokes. Following the sample addition step, wells were sealed and the disc was then inserted into the 3M Integrated Cycler real-time RT-PCR instrument (3M-Focus Diagnostics). Samples were run using pre-programmed conditions set by the manufacturer. Data collection and analysis were performed using Integrated Cycler Studio Software version 4.2. The criteria for valid detection i.e. the positive detection of MC, negative detection of NTC, and the presence of RNA IC amplification curve in negative samples. Samples were considered positive for DENV infection when the Ct value of each serotype was ≤40.0, and ≠ 0. Representative of Simplexa Dengue amplification curves of samples positive for dengue are shown in Figure 1.

### Virus isolation and Envelope gene sequencing

Virus isolation was performed by inoculation of NS1-positive serum samples into C6/36 (*Aedes albopictus*, mid gut) cell line culture [23]. Briefly, a monolayer of cells in T25 flask (Corning, NY, USA) was inoculated with 200 µl of sera in 2 ml of 1X RPMI medium supplemented with 2% of Fetal Bovine Serum (FBS), 2 mM of l-glutamine, 100 U/ml of Penicillin, and 100 µg/ml of Streptomycin (all from Gibco-Life Technologies). Flasks were incubated for 1 hour at 28°C to allow virus attachment. Following the incubation period, inoculation medium was discarded and the medium was replenished with 3 ml of fresh medium. Infected cells were incubated at 28°C for up to 14 days. The presence of virus was confirmed by Envelope gene sequencing, which was performed on DENV RNA extracted from the tissue culture supernatant using method as described previously [19]. Briefly, extracted RNAs were reverse-transcribed into cDNA using Superscript III RT (Invitrogen-Life Technologies). The resulted cDNAs were then used as templates for PCR amplification of the Envelope gene (1,485 nt in length) using *Pfu* Turbo Polymerase (Stratagene-Agilent Technologies, La Jolla, CA, USA). The PCR products were then subjected to sequencing reaction using BigDye Dideoxy Terminator sequencing kits v3.1 (Applied Biosystems) using six overlapping primers described previously [24]. Using this method, we successfully obtained complete sequences of Envelope genes from 40 serum samples (data not shown).

### Data and statistical analysis

All statistical analyses were performed using R statistical software (http://www.r-project.org). To assess the significance of the different results of Simplexa Dengue against the other two methods, we applied McNemar’s test on 2×2 contingency tables derived from the results of each detection method. To assess the significance of infection status and extraction method as factors that might contribute to the performance of each detection method, Wald statistics test on the logistic regression model as implemented in *anova* function of *rms* library from R statistical software was performed [25], [26]. The baseline logistic regression model was *prediction ∼ infection status+extraction method*.

## Results

### Detection rate of Simplexa Dengue compared to conventional RT-PCR and SYBR Green real-time RT-PCR

DENV genome detections were performed in all samples. When tested by conventional RT-PCR method, 53 (28.8%) samples were positive for dengue infection. The SYBR Green real-time RT-PCR detected 81 (44.0%) of dengue-positive samples. Using the same set of samples, Simplexa Dengue detection was employed and demonstrated a significantly increased detection rate in which 141 (76.6%) out of 184 samples were positive for the presence of dengue RNA (Table 1). In all conventional RT-PCR-positive samples (n = 53), one sample was detected as negative by Simplexa Dengue (Positive Percent Agreement = 98.1% (52/53)). In all SYBR Green RT-PCR-positive samples (n = 81), two samples were detected as negative by Simplexa Dengue (Positive Percent Agreement = 97.5% (79/81)).

**Table 1:** Sensitivity of conventional RT-PCR, SYBR Green real-time RT-PCR, and Simplexa Dengue on dengue clinical samples from Indonesia.

Dengue detection	No. of sera tested	No. of positive tests	Sensitivity (%)	95% CI
*On sera confirmed with virus isolation*				
a. Conventional RT-PCR	40	38	95.0	83.1–99.4
b. SYBR Green real-time RT-PCR	40	39	97.5	86.8–99.9
c. Simplexa Dengue	40	40	100.0	91.2–100.0
*On sera confirmed with serology/antigen detection*				
a. Conventional RT-PCR	184	53	28.8	22.4–35.9
b. SYBR Green real-time RT-PCR	184	81	44.0	36.7–51.5
c. Simplexa Dengue	184	141	76.6	69.8–82.5

CI: confidence interval.

Virus isolation is currently the gold/reference standard for dengue detection [10]. To further evaluate the sensitivity of the assays against gold standard, all samples that were positive for NS1 ELISA assay were subjected to virus isolation using the C6/36 cell line and the presence of DENV was confirmed by sequencing of the whole Envelope gene. A total of 40 samples were positive by this method. We then correlated the detection results by conventional RT-PCR, SYBR Green real-time RT-PCR, and Simplexa Dengue assays with the positivity of the gold standard. Of 40 samples confirmed by virus isolation and sequencing, conventional RT-PCR successfully detected 38 samples (sensitivity = 95%), while SYBR Green real-time RT-PCR detected 39 samples (sensitivity = 97.5%) and Simplexa Dengue detected all samples as positive (sensitivity = 100%) (Table 1).

When dengue detections were performed using all evaluated methods in 43 confirmed non-dengue cases and 20 healthy human samples, no dengue virus was detected, which indicated that the specificities of conventional RT-PCR, SYBR Green real-time RT-PCR, and Simplexa Dengue were 100% (Table 2). For all methods, we obtained PPV (positive predictive value) = 1 as there were no false positive result. The negative predictive value (NPV) of RT-PCR, SYBR GREEN real time RT-PCR and Simplexa Dengue were 0.32, 0.38 and 0.59, respectively.

**Table 2:** Specificity of conventional RT-PCR, SYBR Green real-time RT-PCR, and Simplexa Dengue on non-dengue illness and healthy human samples.

Dengue detection	No. of sera tested	No. of negative tests	Specificity (%)	95% CI
*On sera of confirmed non-dengue illness*				
a. Conventional RT-PCR	43	43	100	89.79–100
b. SYBR Green real-time RT-PCR	43	43	100	89.79–100
c. Simplexa Dengue	43	43	100	89.79–100
*On sera of normal healthy donors*				
a. Conventional RT-PCR	20	20	100	79.95–100
b. SYBR Green real-time RT-PCR	20	20	100	79.95–100
c. Simplexa Dengue	20	20	100	79.95–100

CI: confidence interval.

### Performance of the DENV detection methods in relation with the DENV serotypes

To determine how much higher was the sensitivity of Simplexa with respect to each of DENV serotypes, we compared the detection rate of each DENV serotypes for both the conventional RT-PCR and the Simplexa Dengue assays. As shown in Table 3, the detection rate of Simplexa Dengue was higher than that of conventional RT-PCR for all serotypes, except for DENV-3 which had similar detection rate. We did not assess the performance of the SYBR Green real-time RT-PCR in relation with the DENV serotypes because this method was only used for dengue detection and not for serotyping.

**Table 3:** DENV serotype positive detection rate ratio of Simplexa Dengue compared to conventional RT-PCR.

Serotype	Conventional RT-PCR		Simplexa		Positive Detection Rate Ratio^a^
	N	%	N	%	
DENV-1	25	47.2	76	53.9	3.04
DENV-2	6	11.3	14	10.0	2.33
DENV-3	10	18.9	11	7.8	1.1
DENV-4	8	15.1	32	22.6	4
Mix	4	7.5	8	5.7	2
Total	53	100	141	100	2.66

a Positive detection rate ratio was calculated as N _Simplexa_ ÷ N _Conventional RT-PCR._

The presence of concurrent infection of multiple DENV serotypes have been reported in several studies [27]–[31]. In our study, using conventional RT-PCR, we detected four (7.5%) out of 53 RT-PCR-positive samples with mixed infections. When we used the Simplexa Dengue assay, eight out of 141 Simplexa Dengue-positive samples (5.7%) were detected as mixed/concurrent infections (Table 3).

### Performance of the DENV detection methods in relation with infection status and RNA extraction methods

In the sample collection used in this study, we determined the infection status of 184 patients based on IgM and IgG ELISA values. A total of 127 (69.0%) samples were secondary infection, while the rest (57 samples or 31.0%) were primary infection. All three methods detected more positive samples on primary infection samples compared to secondary infection (Table 4). When comparing the detection rates of the evaluated methods against each other, the Simplexa Dengue significantly detected more positive samples in both primary and secondary infection samples compared to other two methods (Table 4).

**Table 4:** Detection rates of evaluated methods in relation with infection status and RNA extraction methods.

Parameter	Conventional		SYBR Green		Simplexa		*p* a	*p* b
	N	%	N	%	N	%		
*Infection status*								
Primary (n = 57)	24	42.1	30	52.6	47	82.5	<0.01	<0.01
Secondary (n = 127)	29	22.8	51	40.2	94	74.0	<0.01	<0.01
*RNA extraction method*								
QIAamp Viral RNA (n = 134)	41	30.6	66	49.3	106	79.1	<0.01	<0.01
MagNA Pure LC (n = 50)	12	24.0	15	30.0	35	70.0	<0.01	<0.01

a McNemar’s Chi-squared test for comparison between conventional RT-PCR and Simplexa.

b McNemar’s Chi-squared test for comparison between SYBR Green RT-PCR and Simplexa.

In this study, we employed two different RNA extraction methods namely the QIAamp Viral RNA Mini Kit (Qiagen) and automated Magna Pure LC (Roche). On RNAs extracted using the above methods, the Simplexa Dengue exhibited significantly higher detection rate (79% and 70%) compared to both conventional RT-PCR (30% and 24%) and SYBR Green real-time RT-PCR (49% and 30%) on samples extracted using QIAamp Viral RNA Mini Kit and automated Magna Pure LC RNA extraction platforms, respectively (Table 4).

To assess whether the infection status and RNA extraction methods affected the detection rate of each evaluated method, we performed ANOVA with logistic regression on the detection results against the two factors as covariates (Table 5). The results indicated that the detection rate of Simplexa Dengue appeared to be not affected by any of those two factors.

**Table 5:** The significance of infection status and extraction method on the performance of DENV detection method.

Parameters	df	Conventional		SYBR Green		Simplexa	
		χ^2^	*p*-value	χ^2^	*p*-value	χ^2^	*p*-value
Infection status	1	7.16	**0.0075**	2.86	0.091	1.69	0.1939
Extraction method	1	1.00	0.3175	5.70	**0.0169**	1.82	0.1768

*p*-value was calculated using Wald statistics on baseline logistic regression model: *detection ∼ extraction method+infection status*, where detection corresponds to the results of conventional RT-PCR, SYBR Green RT-PCR, and Simplexa. *p*-value <0.05 was considered as significant. df: degree of freedom.

## Discussion

Molecular diagnosis of dengue is gradually replacing the traditional method of virus isolation as the gold standard test for viral detection [10], [22], [32]. Various molecular methods are available either as commercial kits or as in-house developed methods with variable sensitivity [10], [11], [17]. In this study, we evaluated the performance of a newly developed real-time RT-PCR detection method in clinical samples. During our molecular surveillance study, samples were previously screened using NS1 and/or IgM and IgG ELISA detection, in which a total of 184 samples were positive for dengue. Using these clinical samples, we assessed the performance of a newly-developed and commercially available Simplexa Dengue assay by comparing the detection rate with two other methods, namely the conventional RT-PCR based on method by Lanciotti et al. [12] and SYBR Green real-time RT-PCR using pan-dengue primers [14]. Using the conventional RT-PCR, we detected DENV in 28.8% of samples (Table 1). The use of SYBR Green real-time RT-PCR increased the detection rate into 44.0%. We then employed the newly developed Simplexa Dengue real-time RT-PCR and obtained a higher detection rate (76.6%). Our evaluation of those three detection methods was further continued using gold standards consisted of sera that were confirmed by virus isolation and Envelope gene sequencing. As expected, detection of DENV genome in sera that were confirmed using the above gold standard yielded higher sensitivity, in which the conventional RT-PCR reached 90%; the SYBR Green real-time RT-PCR reached 97.5%, while Simplexa Dengue reached 100% (Table 1). The specificities of all methods were 100% when tested in 43 non-dengue and 20 healthy human samples (Table 2). Altogether, these findings demonstrated better performance of the Simplexa Dengue assay than that of the conventional RT-PCR and SYBR Green real-time RT-PCR for detection of DENV in the field/surveillance setting.

In our study, one of the clear advantages of using Simplexa Dengue was the higher detection rate compared to conventional and SYBR Green real-time RT-PCR. The higher detection rate of Simplexa Dengue compared to conventional RT-PCR is as expected, since fluorescent-based real-time RT-PCR generally has a better sensitivity than the conventional RT-PCR [11]. Currently, many laboratories in Indonesia are still routinely using conventional RT-PCR method for dengue detection and serotyping, in part because of its simplicity, easy to perform, and does not require expensive equipment. In our laboratory, when this method was used, typically only about 20–30% of dengue-suspected samples collected were positive for DENV genome (Sasmono et al., unpublished). Simplexa Dengue presented almost a three-fold increase in the number of DENV-positive samples (in this study reached 76.6%) and also serotyped them simultaneously. This high detection rate will be very useful in epidemiologic surveillance, especially in Indonesia where data on DENV serotype distribution is scarce. Moreover, this method offers rapid detection in which results can be obtained within one hour, compared to conventional RT-PCR which requires about two to three hours for the results.

The lower detection rates of conventional and SYBR Green real-time RT-PCR might be attributed to the primers used in those two methods. The conventional RT-PCR method was developed more than two decades ago. The DENV possesses high rate of mutation due to the lack of proof-reading RNA polymerase which is typical of other RNA viruses [33]. The mutations that occurred in the DENV genomes may cause nucleotide mismatches with the primers used in the PCR detection. The primers used in conventional RT-PCR method [12] have been modified to increase the sensitivity [22], [34]. To assess the identity of the original conventional RT-PCR primers [12] with the DENV genome sequences, we aligned the nucleotide sequence of D1 and D2 primers, which are located in the Capsid-prM genes, with 86 DENV genomes of all serotypes from Indonesia and other countries. We observed 21 different patterns of mismatches (Table S1A). The mismatches were also occurred in one nucleotide position corresponds to modified D1 primer described previously [34]. For the SYBR Green real-time RT-PCR, alignment was also performed on primers located in the 3′UTR region [14], in which 9 mismatch patterns were observed in the forward pan-dengue primer (Table S1B). Altogether, these nucleotide mismatches, which can reduce the efficiency of the primers to bind their genomic target sequences, may underlie the lower sensitivity of both conventional RT-PCR and SYBR Green real-time RT-PCR compared to Simplexa Dengue assay. We were not able to align the Simplexa Dengue primers with the DENV genomes as the primer sequences were not publicly available. Based on the manufacturer’s information, the binding sites of the Simplexa Dengue primers were in the NS5, NS3, NS5, and capsid genes for DENV-1, DENV-2, DENV-3, and DENV-4, respectively.

To assess the performance of Simplexa Dengue in detecting different DENV serotypes in clinical samples, we analyzed our serotype data and linked them with the detection rates. We observed higher positive detection rate ratios of Simplexa Dengue for all serotypes, except for DENV-3 which was quite similar with the conventional RT-PCR detection rate (Table 3). A study by Tricou *et al.* [35] described the serotype-specific viremia kinetics and NS1 levels in dengue patients, in which DENV-1 exhibited a relatively higher viremia than DENV-2. The different viremia levels of particular DENV serotypes may have implications in the sensitivity of dengue molecular assays. We were not sure whether DENV-3 strains in our study indeed possessed higher viremia levels compared to other DENV, which might account for similar detection rates of both conventional RT-PCR and Simplexa Dengue. Measurement of viremia levels in patients infected by DENV would be beneficial to confirm this. The higher detection rate of Simplexa Dengue suggested that the method has lower detection limit than conventional RT-PCR but this need to be confirmed using a Limit of Detection study for all four serotypes.

We observed a higher detection rate of DENV-4 identified by the Simplexa Dengue assay than conventional RT-PCR (Table 3). Currently, we are not sure why the Simplexa Dengue assay is more sensitive for DENV-4 than conventional RT-PCR. It is possible that the conventional RT-PCR primers by Lanciotti et al. [12] did not match with the currently circulating virus strains and thus decreased sensitivity for detecting DENV-4. Indeed, we observed one nucleotide mismatch in the forward primer and two-to four nucleotide mismatches in the reverse primers compared to the genome sequences of eight Indonesian DENV-4 strains (unpublished results). However, when new primers were generated and used to re-PCR the RT-PCR-negative DENV-4 samples, the detection rate was not increased.

In our study, using conventional RT-PCR we detected four concurrent infections with more than one dengue serotype (Table 3). The Simplexa Dengue detected eight samples as concurrent infections. Concurrent infections of multiple dengue serotypes have been reported in Indonesia, Mexico, and Puerto Rico [36] as well as other countries [29], [31], [37], [38]. During epidemics in those countries, DENV concurrent infections were detected in about 5.5% of the samples [36]. In our study, a little higher percentage of concurrent infection was observed and most of them involved infection of DENV-1. This phenomenon was possible because during the surveillance period (2010–2012), DENV-1 was prominent in many Indonesian cities studied. For example, we observed the predominance of DENV-1 (35.5%) in Semarang city [19], and similar predominance was also observed in other eight cities (Sasmono et al., manuscript in preparation). The proportion of DENV serotypes in samples used in this study also clearly demonstrated the predominance of DENV-1 (Table 3). We were able to confirm the presence of concurrent/multiple DENV serotypes infection in two samples using virus isolation and Envelope gene sequencing, including determining the genotypes of the infecting viruses (data not shown). The possibility of cross-contamination generating the detection of concurrent infections cannot be ruled out. However, in our study, we employed strict measures to prevent cross-contamination by processing the samples in dedicated containments/rooms and using separate equipment for each step of the extraction and amplification (single directional flow system). Furthermore, our laboratory also followed the quality assurance program that was necessary for laboratories that performed and offered services for diagnosis for dengue [6]. Overall, our data suggest the presence of concurrent DENV infections in hyperendemic region such as Indonesia, in which all DENV serotypes are currently circulating.

Our data showed that not all samples that were antigenically or serologically positive for dengue were positive by all RT-PCR methods evaluated, although the samples were collected in the early stage of the disease. This is understandable since serological tests are generally more sensitive than the RT-PCR. Previous studies have described the difficulty of virus detection in the presence of neutralizing antibody [39], which is one of the characteristics of secondary infection. Our sample collection was dominated by secondary infection (69.0%). Therefore, the lower detection rates of RT-PCR compared to serological tests was likely. Our detection methods also exhibited higher detection rates in primary infection compared to secondary infection (Table 4). This is in accordance with previous studies that observed higher levels of viremia in primary versus secondary infection and earlier and faster clearance of viremia in secondary infection [35], [40]. The high levels of viremia in primary infection facilitated the higher sensitivity of the assays in detecting DENV RNA genome.

In terms of the DENV RNA extraction method, we compared two extraction protocols. The first was manual extraction using the affinity column produced by Qiagen (QIAamp Viral RNA Mini Kit) and the second one was automated extraction using the MagNA Pure LC (Roche) extraction systems. Using both methods, Simplexa Dengue had higher detection rate compared to both SYBR Green and conventional RT-PCR (Table 4). Although the proportion of the positively detected samples appeared to be higher in QIAamp compared to MagNA Pure extracted samples, RNA extraction methods were not a factor that influenced Simplexa Dengue detection rate (Table 5). This finding also supports previous report about the comparability of Qiagen and MagNA Pure extraction methods on the yield and purity of the extraction products [41]. The use of an automated extraction method will be beneficial especially when processing a large number of samples, for example in outbreak settings, although the disadvantage will be the need to provide expensive equipment. In term of consumables/reagents cost effectiveness, we observed that the cost of automated RNA extraction per sample is similar to manual extraction (data not shown). For Simplexa Dengue usage, this finding suggests the compatibility of this new DENV detection method with RNA obtained from either manual or automated extraction.

The Simplexa Dengue method employs the use of an internal control (IC) to monitor the validity of the nucleic acid extraction, a positive molecular control (MC) and no template control (NTC). The assay combines reverse transcription and PCR detection in single reaction which greatly decreases the test’s duration (performed within one hour) and the risks of contamination. Furthermore, this method applies automated amplification and data analysis processes as well as using assay’s specific and dedicated reagents. Altogether, these features minimize the involvement of humans during the detection process which reduces the possibility of human errors. Combined with an automated nucleic acid extraction system, which is also rapid and minimizes the human involvement during extraction process, these methods will be suitable for dengue detection in various settings such as epidemiological surveillance, clinical management, dengue research, and vaccine trials. Of particular interest, the dengue vaccine clinical trials require the confirmation of dengue cases in vaccinated individuals to determine the vaccine efficacy and for the early detection of vaccine-escape mutants [11]. The high sensitivity and specificity of Simplexa Dengue for dengue detection will be beneficial for rapid and accurate diagnosis of dengue infection in various settings.

## Supporting Information

Table S1 A. Alignment of conventional RT-PCR primers based on Lanciotti et al (1992) with DENV genome sequences from Indonesia and other countries; B. Alignment of Pan-dengue RT-PCR primer based on Lai et al (2007) with DENV genome sequences from Indonesia and other countries; C. GenBank accession number of DENV genomes used in primer alignments.
(PDF)
