# Supplementary material for: Performance of Simplexa Dengue Molecular Assay Compared to Conventional and SYBR Green RT-PCR for Detection of Dengue Infection in Indonesia
Source: PLoS One. 2014 Aug 7;9(8):e103815. doi: 10.1371/journal.pone.0103815 (PMC4125142; doi:10.1371/journal.pone.0103815)
Supplement: Table S1 — A. Alignment of conventional RT-PCR primers based on Lanciotti et al (1992) with DENV genome sequences from Indonesia and other countries; B. Alignment of Pan-dengue RT-PCR primer based on Lai et al (2007) with DENV genome sequences from Indonesia and other countries; C. GenBank accession number of DENV genomes used in primer alignments. (PDF) [file pone.0103815.s001.pdf]

A. Alignment of conventional RT-PCR primers based on Lanciotti et al (1992) with DENV genome sequences from Indonesia and other countries

	D1 forward primer sequence	D2 reverse primer sequence	Country of origin & isolate number											
	T C A A T A T G C T G A A A C G C G C G A G A A A C C G	T T G C A C C A A C A G T C A A T G T C T T C A G G T T C	ID	SG	TH	PH	TL	VN	AU	BN	MY	BR	VE	MX
Pattern 1	. . . .	. . . . C . . . A . . T	3	1	1	-	-	-	-	1	1	-	-	-
Pattern 2	. . . .	. . . . A . . . . C . . . A . . T	-	3	1	-	-	4	-	-	-	-	-	-
Pattern 3	. . . .	. . . . G . . A . . . . C A . . A . . T	-	-	1	-	-	-	-	-	-	-	-	-
Pattern 4	. . . .	. . . . G . . . . C . . . A . . T	-	-	-	-	-	-	-	-	-	2	2	2
Pattern 5	. . . . T	. . A . . . . . G	-	-	-	-	-	-	-	-	-	-	2	-
Pattern 6	. . . . T	. . . . G . . . . A . . . . . G	-	-	-	-	-	-	-	-	-	-	-	2
Pattern 7	. . . . T	. . . . G	-	1	2	-	-	-	-	-	-	2	-	-
Pattern 8	. . . . T	. . . . G . . . . C . .	12	2	-	1	-	-	-	-	-	-	-	-
Pattern 9	. . . . T	. . . . G . . . . C . . . . . C . .	-	-	-	-	4	-	-	-	-	-	-	-
Pattern 10	. . . . T	. . . . C . .	1	-	-	1	-	-	-	-	-	-	-	-
Pattern 11	. . . . A	. . . . G	-	-	2	-	-	-	-	-	-	-	-	-
Pattern 12	. . . . A	. . . . G . . A	1	1	1	-	-	-	-	-	-	2	2	-
Pattern 13	. . . . A	. . . . G . . A . . . . G	-	-	3	-	-	-	-	-	-	-	-	-
Pattern 14	. . . . A	. . . . G . . A . . . . C	-	-	-	1	-	-	-	-	-	-	-	-
Pattern 15	. . . . A	. . . . G . . A . . . . C . .	-	-	-	-	-	-	-	-	1	-	-	-
Pattern 16	. . . . A	. . . . A . . T . . . . T	-	-	-	-	-	-	-	-	-	2	2	2
Pattern 17	. . . . A	. . . . T . . . . T	5	3	-	-	-	1	-	1	-	-	-	-
Pattern 18	. . . . A	. . . . T . . . . T . . C . .	-	-	1	-	-	-	-	-	-	-	-	-
Pattern 19	. . . . A	. . . . A . . T . . . . T . . C . .	-	-	1	-	-	-	-	-	-	-	-	-
Pattern 20	. . . . A	. G . . . . . C . . . . . . T . . . . T	-	-	-	-	-	-	1	-	-	-	-	-
Pattern 21	. . . . A	. . A . . . . . G . . A . . T . . . . T	-	-	1	-	-	-	-	-	-	-	-	-

Country abbreviations: Indonesia (ID), Singapore (SG), Thailand (TH), Philippine (PH), Timor Leste (TL), Vietnam (VN), Australia (AU), Brunei (BN), Malaysia (MY), Brazil (BR), Venezuela (VE), Mexico (MX).

B. Alignment of Pan-dengue RT-PCR primer based on Lai et al (2007) with DENV genome sequences from Indonesia and other countries

	Pan-dengue forward primer sequence	Country of origin & isolate number											
	T T G A G T A A A C Y R T G C T G C C T G T A G C T C	ID	SG	TH	PH	TL	VN	AU	BN	MY	BR	VE	MX
Pattern 1	. . . . . C . . . . G	1	-	1	-	-	-	-	-	-	-	-	-
Pattern 2	. . . . . C . . . . T . . .	-	1	-	-	-	-	-	-	-	-	-	-
Pattern 3	. . . . . C	13	7	9	3	4	4	-	-	2	4	4	2
Pattern 4	. . . . A	5	3	1	-	-	1	-	1	-	2		2
Pattern 5	. . . . . C . . . . A	-	-	2	-	-	-	-	-	-	-	2	-
Pattern 6	. . . . A . . . . T	-	-	-	-	-	-	1	-	-	-	-	-
Pattern 7	. . . . . C . . G	1	-	-	-	-	-	-	1	-	-	2	-
Pattern 8	. . . . . C . . G . . . . G	-	-	1	-	-	-	-	-	-	2	-	2
Pattern 9	. C . . . C . . G	1	-	-	-	-	-	-	-	-	-	-	-

Country abbreviations are as above.

C. GenBank accession number of DENV genomes used in primer alignments

No	Accession No	Country	Year	Serotype
1	JX669474	Brazil	2001	DENV-1
2	JX669475	Brazil	2002	DENV-1
3	EU179861	Brunei	2006	DENV-1
4	AB189120	Indonesia	1998	DENV-1
5	AB189121	Indonesia	1998	DENV-1
6	AY858983	Indonesia	2004	DENV-1
7	EF457905	Malaysia	1972	DENV-1
8	KJ189369	Mexico	2011	DENV-1
9	KJ189368	Mexico	2012	DENV-1
10	EU081235	Singapore	2005	DENV-1
11	EU081238	Singapore	2005	DENV-1
12	EU081254	Singapore	2005	DENV-1
13	EU081280	Singapore	2006	DENV-1
14	AF180817	Thailand	1963	DENV-1
15	AY732476	Thailand	1980	DENV-1
16	AY732482	Thailand	2001	DENV-1
17	HQ332182	Venezuela	2006	DENV-1
18	HQ332183	Venezuela	2007	DENV-1
19	EU249491	Vietnam	2006	DENV-1
20	EU482525	Vietnam	2006	DENV-1
21	EU482540	Vietnam	2006	DENV-1
22	EU482707	Vietnam	2006	DENV-1
23	AY037116	Australia	1993	DENV-2
24	JX669487	Brazil	2000	DENV-2
25	JX669488	Brazil	2002	DENV-2
26	EU179857	Brunei	2005	DENV-2
27	AB189122	Indonesia	1998	DENV-2
28	AB189123	Indonesia	1998	DENV-2
29	AB189124	Indonesia	1998	DENV-2
30	AY858035	Indonesia	2004	DENV-2
31	AY858036	Indonesia	2004	DENV-2
32	KJ189311	Mexico	2007	DENV-2
33	KJ189370	Mexico	2011	DENV-2
34	EU081177	Singapore	2005	DENV-2
35	EU081178	Singapore	2005	DENV-2
36	EU081179	Singapore	2005	DENV-2
37	U87411	Thailand	1984	DENV-2
38	DQ181801	Thailand	1990	DENV-2
39	AF100462	Thailand	1995	DENV-2
40	HQ332190	Venezuela	2007	DENV-2
41	HQ332189	Venezuela	2007	DENV-2
42	EU482640	Vietnam	2006	DENV-2
43	JF808127	Brazil	2002	DENV-3
44	JF808126	Brazil	2003	DENV-3
45	AY648961	Indonesia	1978	DENV-3
46	AY858038	Indonesia	1988	DENV-3
47	AY858039	Indonesia	1998	DENV-3
48	AB189125	Indonesia	1998	DENV-3
49	AB189126	Indonesia	1998	DENV-3
50	AB189127	Indonesia	1998	DENV-3
51	AB189128	Indonesia	1998	DENV-3
52	AY858037	Indonesia	2004	DENV-3
53	AY858040	Indonesia	2004	DENV-3
54	AY858041	Indonesia	2004	DENV-3
55	AY858042	Indonesia	2004	DENV-3
56	AY858043	Indonesia	2004	DENV-3
57	AY858044	Indonesia	2004	DENV-3
58	FJ898441	Mexico	2006	DENV-3
59	FJ898442	Mexico	2007	DENV-3
60	M93130	Philippines	1956	DENV-3
61	AY496879	Philippines	1997	DENV-3
62	AY766104	Singapore	1995	DENV-3
63	EU081221	Singapore	2005	DENV-3
64	EU081223	Singapore	2005	DENV-3
65	AY876494	Thailand	1994	DENV-3
66	AY923865	Thailand	1994	DENV-3
67	AB214879	Timor Leste	2005	DENV-3
68	AB214880	Timor Leste	2005	DENV-3
69	AB214881	Timor Leste	2005	DENV-3
70	AB214882	Timor Leste	2005	DENV-3
71	HQ332171	Venezuela	2006	DENV-3
72	HQ332170	Venezuela	2006	DENV-3
73	JN559741	Brazil	2010	DENV-4
74	JN983813	Brazil	2010	DENV-4
75	AY858050	Indonesia	2004	DENV-4
76	EF457906	Malaysia	1975	DENV-4
77	AY947539	Philippines	1956	DENV-4
78	AY762085	Singapore	1995	DENV-4
79	AY618991	Thailand	1977	DENV-4
80	AY618990	Thailand	1991	DENV-4
81	AY618988	Thailand	1997	DENV-4
82	AY618989	Thailand	1997	DENV-4
83	AY618993	Thailand	2000	DENV-4
84	AY618992	Thailand	2001	DENV-4
85	HQ332176	Venezuela	2007	DENV-4
86	HQ332175	Venezuela	2007	DENV-4
